# Increased Chondrocyte Apoptosis Is Associated with Progression of Osteoarthritis in Spontaneous Guinea Pig Models of the Disease

**DOI:** 10.3390/ijms140917729

**Published:** 2013-08-29

**Authors:** Zaitunnatakhin Zamli, Michael A. Adams, John F. Tarlton, Mohammed Sharif

**Affiliations:** 1 Centre for Comparative and Clinical Anatomy, University of Bristol, Southwell Street, Bristol BS2 8EJ, UK; E-Mails: zaitun.zamli@bristol.ac.uk (Z.Z.); m.a.adams@bristol.ac.uk (M.A.A.); 2 School of Veterinary Science, University of Bristol, Bristol BS8 1TH, UK; E-Mail: john.tarlton@bristol.ac.uk

**Keywords:** chondrocyte apoptosis, osteoarthritis, animal models, disease progression, caspase-3

## Abstract

Osteoarthritis (OA) is the most common joint disease characterised by degradation of articular cartilage and bone remodelling. For almost a decade chondrocyte apoptosis has been investigated as a possible mechanism of cartilage damage in OA, but its precise role in initiation and/or progression of OA remains to the determined. The aim of this study is to determine the role of chondrocyte apoptosis in spontaneous animal models of OA. Right tibias from six male Dunkin Hartley (DH) and Bristol Strain 2 (BS2) guinea pigs were collected at 10, 16, 24 and 30 weeks of age. Fresh-frozen sections of tibial epiphysis were microscopically scored for OA, and immunostained with caspase-3 and TUNEL for apoptotic chondrocytes. The DH strain had more pronounced cartilage damage than BS2, especially at 30 weeks. At this time point, the apoptotic chondrocytes were largely confined to the deep zone of articular cartilage (AC) with a greater percentage in the medial side of DH than BS2 (DH: 5.7%, 95% CI: 4.2–7.2), BS2: 4.8%, 95% CI: 3.8–5.8), *p* > 0.05). DH had a significant progression of chondrocyte death between 24 to 30 weeks during which time significant changes were observed in AC fibrillation, proteoglycan depletion and overall microscopic OA score. A strong correlation (*p* ≤ 0.01) was found between chondrocyte apoptosis and AC fibrillation (*r* = 0.3), cellularity (*r* = 0.4) and overall microscopic OA scores (*r* = 0.4). Overall, the rate of progression in OA and apoptosis over the study period was greater in the DH (*versus* BS2) and the medial AC (*versus* lateral). Chondrocyte apoptosis was higher at the later stage of OA development when the cartilage matrix was hypocellular and highly fibrillated, suggesting that chondrocyte apoptosis is a late event in OA.

## 1. Introduction

Chondrocyte death is a known feature of osteoarthritis (OA). It has been hypothesised as a key player in the pathogenesis of OA based on the presence of hypocellularity and a large number of empty lacunae, especially in regions close to fibrillated areas of osteoarthritic cartilage [1,2]. Previous studies have shown that the number of chondrocytes decreases with severity of the disease [1,3,4] and aging [5–9], and that these changes are associated with the production of reactive oxygen species (ROS), a lack of growth factors, release of glycosaminoglycan (GAG) and mechanical injury [10–12]. Chondrocyte death by apoptosis [3,13], necrosis [14] or other cell death mechanisms such as chondroptosis [15,16], lead to loss of resident cells in the articular cartilage resulting in unfavourable consequences such as impairmed matrix remodelling and development of OA, even when the biochemical and/or biomechanical demands in the joint are normal.

Chondrocyte death by apoptosis has been reported both in human [13,17–20] and in animal models of OA [3,9,21–23]. In these studies, chondrocyte apoptosis was detected by caspase-3 expression and TUNEL, and confirmed by electron microscopic examination. In *in vitro* studies, chondrocyte apoptosis can be induced by exposing the normal cartilage explants or chondrocyte cultures either to biological [14,19,24] (e.g., nitric oxide (NO), collagenase, anti-CD95) or mechanical factors [10,11,25,26] (e.g., shear strain, loading strain). This effect can be inhibited by treatment with caspase inhibitor [24,27,28] (e.g., z.VAD.fmk), Insulin-like growth factor 1 (IGF-1) [24], *N*-acetylcysteine (NAC) [29] and p38 MAPK inhibitor [14]. All of these studies support our view that chondrocyte apoptosis is largely responsible for hypocellularity in articular cartilage and may therefore play an important role in cartilage loss and OA [18]. However, the degree of chondrocyte loss in OA cartilage is inconsistent between studies [3,13,19,30,31], and this disagreement may be dependent on the nature of study (e.g., primary *vs.* secondary OA, induced *vs.* spontaneous animal models of OA), the stage of the disease or types of analysis being used. Moreover, as we reviewed recently [32] the important question of whether chondrocyte apoptosis is a cause or consequence of cartilage degradation needs to be addressed properly in a suitable model. Thus, there is a need for longitudinal studies of suitable animal models of OA to clarify the role of chondrocyte apoptosis in the pathogenesis of OA.

The Dunkin Hartley (DH) guinea pig is one of the most widely used strains for spontaneous animal model of OA since their histological and biochemical changes resemble that of human OA [33]. Since chondrocyte apoptosis is a part of normal physiological process in aging, a suitable control group is required in order to distinguish changes due to aging or pathology. Currently, the available control strains for DH are Strain 13 [34], Weiser-Maple [35] and Bristol strain 2 (BS2) [36–38]. There were only three studies that have used the latter strain as a control, and of these studies, only two described the histological changes of cartilage in this animal [36,38]. The overall aim of the present study is to determine the role of chondrocyte apoptosis in the initiation and progression of OA development in DH and to validate the use of BS2 as a control for this animal model. A further aim of the study is to test the hypothesis that chondrocyte apoptosis is an early phenomenon in cartilage damage and development of OA.

## 2. Results

### 2.1. Body Weight

**Figure 1:**
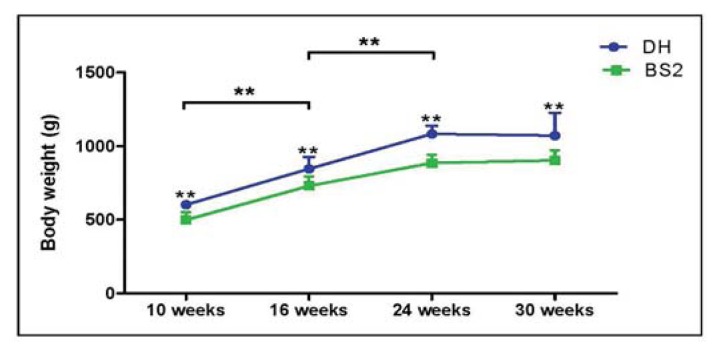
Body weight of Dunkin Hartley (DH) (*n* = 24) and Bristol Strain 2 (BS2) (*n* = 24) over 30 weeks study period. Error bars represent the 95% CI.

DH guinea pigs were significantly heavier than BS2 by an average of 19% (*p* < 0.01) (Figure 1). However, over time, both strains had a similar rate of growth and showed a dramatic increase of body weight between 10 (DH: 601.8 g (95% CI: 579.4–624.3); BS2: 498.3 g (95% CI: 446.3–550.4)) and 24 (DH: 1083.3 g (95% CI: 1028.2–1138.4); BS2: 885.8 g (95% CI: 829.5–942.2)) weeks of age as expected before plateauing at later time points.

### 2.2. Histopathological Changes in AC

**Figure 2:**
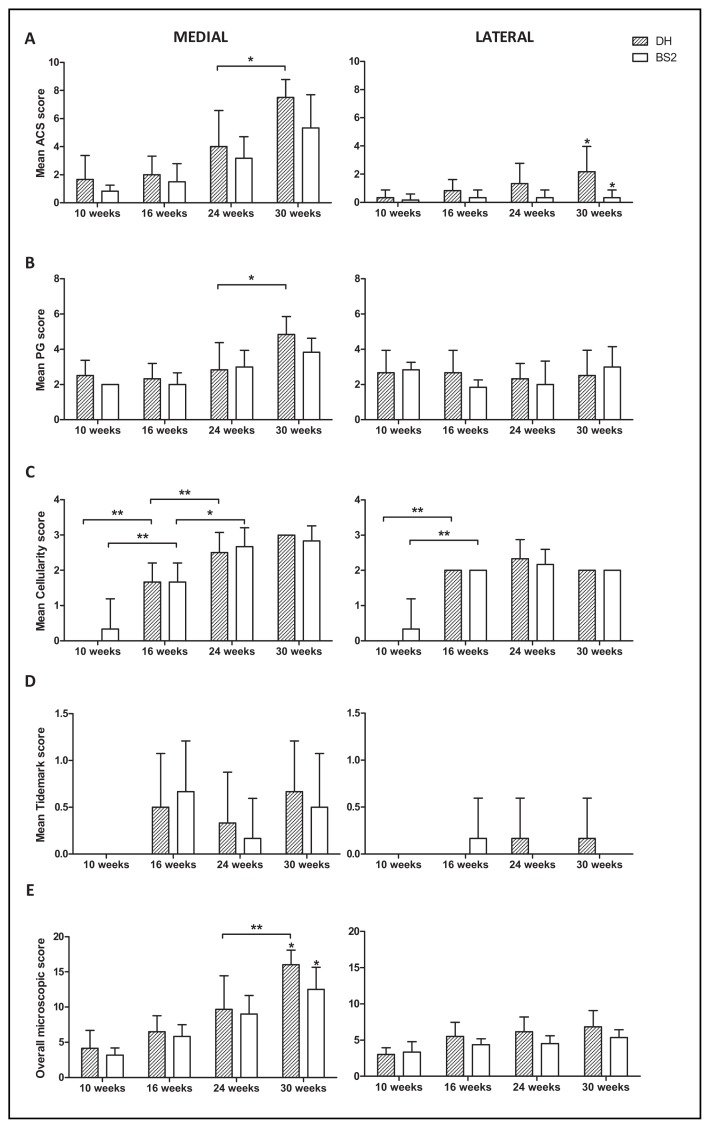
Microscopic score of the medial and lateral side of DH and BS2 tibial plateau at four different time points. Mean of articular cartilage surface (ACS) scores (**A**); proteoglycan (PG) (**B**); cellularity (**C**); tidemark (**D**) and overall microscopic score (**E**) were compared between strains (DH: *n* = 6; BS2: *n* = 6 at each time point) and adjacent time points. A significant difference of *p* ≤ 0.05 and *p* ≤ 0.01 was denoted as * and **, respectively. Error bars represent the 95% CI.

Articular cartilage surface (ACS) score was higher in the DH strain than BS2 at all time points in both medial and lateral (Figure 2A), but reached statistical significance only in the lateral side of AC at 30 weeks. Microscopic changes of AC (*i.e.*, PG loss, focal cell loss *etc.*) in DH and BS2 were observed as early as 10 weeks of age. These changes progressed with age and were more pronounced in the medial than the lateral side of AC. Overall there was no significant difference between strains in any components of microscopic scores (Figure 2A–D). DH had a slightly higher overall microscopic score than BS2 at all time points in both medial and lateral, but reached statistical significance only in the medial side of AC at 30 weeks (DH: 16.0 (95% CI: 13.9–18.1), BS2: 12.5 (95% CI: 9.3–15.7), *p* ≤ 0.05, Figure 2E). Across the time points, both strains had a significant increase of cellularity score in the medial and lateral side of AC between 10 and 16 weeks (*p* ≤ 0.01). This trend continued to increase up to 24 weeks in the medial side of both strains. Following the cellularity changes, only the medial side of DH had a significant progression of AC fibrillation (*p* ≤ 0.05), PG loss (*p* ≤ 0.05) and overall microscopic score (*p* ≤ 0.01) between 24 and 30 weeks of age.

### 2.3. Chondrocyte Apoptosis

**Figure 3:**
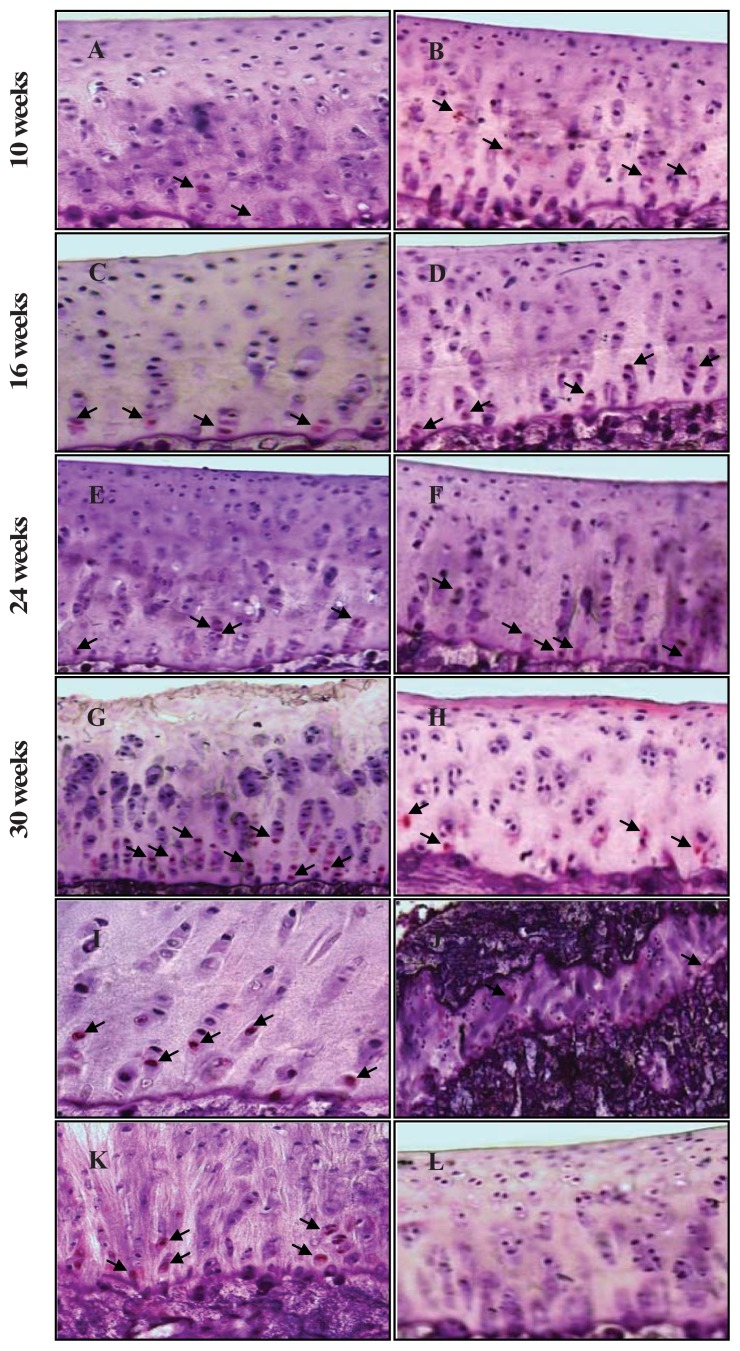
Representative sections of the medial side of right tibial epiphysis stained with/without caspase-3. Sections stained with caspase-3 antibody at four different time points (DH: **A**, **C**, **E** and **G**; BS2: **B**, **D**, **F** and **H**); a higher magnification (**I**); the growth plate (**J**) and fibrocartilage of anterior cruciate ligament (**K**) show caspase-3 positive cells (red cytoplasmic staining; arrow). A negative control section is shown in (**L**).

Figure 3 shows the histological images of caspase-3 staining in the medial side of AC from both animal strains at the four time points. At higher magnification (×400), the caspase-3 positive cells showing red staining and negative control showing no staining in the cytoplasm are easy to visualize. Apoptotic chondrocytes were found in all AC zones and most pronounced in the deep zone as the disease progressed. Interestingly, caspase-3 positive cells were also observed in the tidemark region, the growth plate, and in the fibrocartilage of the anterior cruciate ligament (ACL) (Figure 3I–K, respectively).

**Figure 4:**
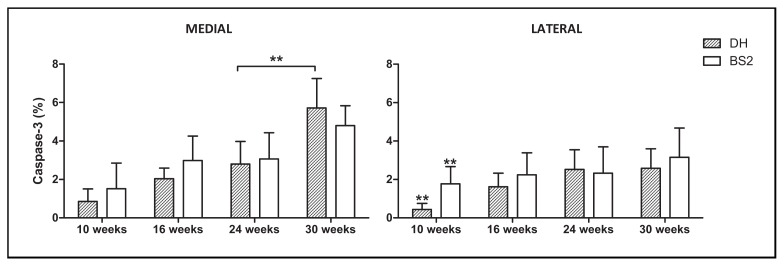
Percentage of caspase-3 positive chondrocytes in the medial and lateral side of AC in DH (*n* = 6) and BS2 (*n* = 6) at four different time points. Error bars represent the 95% CI.

The percentage of caspase-3 positive chondrocytes was always higher in the medial (DH: 2.9% (95% CI: 2.0–3.7); BS2: 3.1% (95% CI: 2.4–3.8)) than the lateral side (DH: 1.8% (95% CI: 1.3–2.3); BS2: 2.4% (95% CI: 1.9–2.9)) and increases gradually over time in both strains (Figure 4). In the medial side, BS2 showed a slightly higher percentage of chondrocyte apoptosis than DH for the earlier time points, but the rate of increase of apoptotic cells was greater in the DH such that at 30 weeks, the DH was greater than the BS2 [DH: 5.7% (95% CI: 4.2–7.2), BS2: 4.8% (95% CI: 3.8–5.8), *p* > 0.05]. Though the increase in apoptotic chondrocytes was apparent with increasing age, it was only in the medial side of the DH AC between 24 and 30 weeks that this was significant (*p* ≤ 0.01). A significant difference between strains at a particular time point is only seen in the lateral side of AC at 10 weeks (*p* ≤ 0.01), with the BS2 greater than the DH.

**Figure 5:**
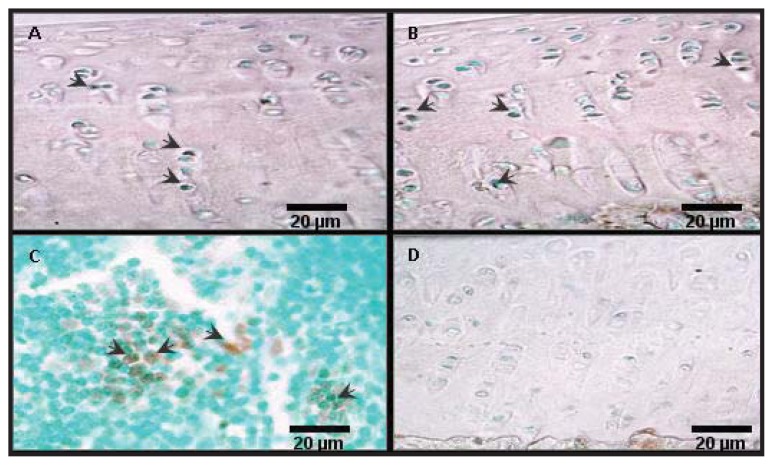
Representative sections from TUNEL. Sections stained with TUNEL at 10 weeks (**A**) and 30 weeks of age (**B**) are shown. Positive and negative control sections are shown in (**C**) and (**D**), respectively. TUNEL positive cells are indicated with the arrows.

TUNEL on selected sections confirmed the localisation of apoptotic chondrocytes found in caspase-3 staining (Figure 5). The percentage of chondrocyte apoptosis detected by TUNEL was always higher than with the caspase-3 method, but the correlation between the two techniques was statistically significant (*r* = 0.7, *p* ≤ 0.01).

### 2.4. Correlation between Chondrocyte Apoptosis and AC Degradation

Chondrocyte apoptosis was significantly correlated (*p* ≤ 0.01) with AC fibrillation, cellularity and overall microscopic scores (Spearman’s partial correlation (adjusted for age, strain and weight), *r* = 0.3, 0.4 and 0.4, respectively).

## 3. Discussion

We report a number of important findings in the development and progression of OA in two different strains of guinea pigs previously found to be susceptible (DH) and resistant (BS2) to the progression of spontaneous OA [38]. The medial side of the tibial plateau was more prone to disease than the lateral side, not only in DH but also in the BS2. These changes progressed with age and correlated with levels of chondrocyte apoptosis. Also, chondrocyte apoptosis was higher in the medial cartilage and largely localised to the deep zone (DZ) in both strains. Moreover, in the DH animals, body weight appears to be a risk factor of progression in chondrocyte apoptosis and cartilage damage, and chondrocyte apoptosis appear to follow changes in cartilage cellularity.

Our data demonstrating that the medial side of tibial plateau had greater cartilage damage than the lateral side in DH is consistent with previous studies of this strain of guinea pig [33,39–44]. Interestingly, we also found the same pattern of disease in the BS2 strain, used in several studies as a control for OA in DH. In a study of human knee joints, it was found that patients who suffered from anterior cruciate ligament (ACL) deficiency with varus alignment tend to have a higher medial tibiafemoral compartment load [45]. Thus, our data may indicate that in the guinea pig models of OA, the weight bearing has shifted and induced extra load onto the medial side during the course of the disease. Shifting of the joint alignment towards varus could be as a result of knee joint instability during development of OA in this guinea pig model. Knee joint instability has been reported following trauma, musculotendinous and ligamentous weaknesses or joint surface incongruity [46]. Moreover, Quasnichka *et al.* [37] reported that the ACL of DH was abnormally remodelled and the knee joint was unstable prior to the macroscopic changes of the AC. Interestingly, in the present study we found that there was chondrocyte apoptosis in the fibrocartilage of ACL which may have contributed to abnormal remodelling and laxity of the knee joint. Taken together, the unstable knee joint may cause a varus adaptation and subsequently trigger the chondrocytes to compensate for changes in compressive stress by increasing matrix turnover. Failing to compensate for changes in load may lead to chondrocyte apoptosis and degradation of the AC in the medial side.

Our observation that chondrocyte apoptosis is higher in the medial side and largely localised in the deep zone (DZ) of AC supports other studies of spontaneous OA, both in guinea pigs [23] and some human studies [19], but differs considerably from results of studies where OA was induced. Thus, animal models of surgical/injury-induced OA (*i.e.*, ACL transection, menisectomy, mechanical compression) showed that chondrocyte apoptosis was mainly confined to the superficial (SZ) and middle zone (MZ) [3,22,25,30,47]. These variations may reflect the different underlying triggering mechanisms of cell death in OA cartilage. Chen and co-workers [48] found that chondrocytes in the SZ were the most susceptible to mechanical loading due to the lack of GAG and parallel arrangement of type II collagen in this region. However, at a greater stress (>20 MPa) and a higher stress rate (>60 MPa/s), chondrocytes in the DZ were dying by apoptosis [49–51]. Similarly, excessive load on cartilage that lost its stiffness, following immersion in a hypertonic contrast agent, lead to greater chondrocyte death at the DZ [25]. Thus it may be that in our studies, the extra loading is not directly linked to chondrocyte death in the DZ of a spontaneous animal model of OA. Alternatively, one could argue that the presence of chondrocyte apoptosis in the tidemark region of DH is due to continuous remodelling in the calcified cartilage during varus loading. The latter hypothesis is supported by Adams *et al.* [9] who demonstrated that a significant number of apoptotic chondrocytes were present in the calcified cartilage of Wistar rat and C57BL mouse that developed OA spontaneously. Furthermore, thickening of the medial subchondral bone plate was also observed during the development of OA [52], which suggest that chondrocyte apoptosis in the DZ and the calcified cartilage may be at least partly involved in early bone changes in OA.

Across the time points, DH and BS2 showed typical microscopic changes of osteoarthritic cartilage in a time-dependent manner. Both strains had a significant increase of cellularity score in the medial and lateral side of AC between 10 to 16 weeks of age, and only the medial side continued to show changes at the later time points (16–24 weeks). This implies that early changes in cellularity represent normal ageing and responses to increased body weight, and that continued changes beyond 16 weeks, confined to the medial side of AC, represent OA related changes. Moreover, a significant progression of chondrocyte apoptosis with a dramatic increase of AC fibrillation, PG loss and overall microscopic score was noticed only in the medial side of DH between the final time points. Since DH and BS2 had a similar rate of growth and weight gain, the observed changes are likely due to pathology rather than growth or aging. Furthermore, since the rate of apoptosis was only significantly higher when the extracellular matrix was hypocellular and highly fibrillated, chondrocyte death by apoptosis may be a late event in the spontaneous animal model of OA. This contention is supported by a study by Mistry *et al.* [1] who showed that chondrocyte apoptosis was increased following AC fibrillation and suggested that chondrocyte death by apoptosis is a consequence of other AC changes in the STR/ort mouse model of OA. Overall, the above findings suggest that chondrocytes play an important role in AC degradation and that chondrocyte apoptosis is more important in the progression rather than initiation of OA.

When comparing between the medial side of DH and BS2, the overall microscopic score showed that there was no significant difference in AC damage between strains other than at 30 weeks, at which time both strains had AC fibrillation at least up to the middle zone (MZ). This finding appears contradictory to the previous studies in which BS2 did not show any fibrillation at least until 36 weeks of age [36,38]. Although both our study and the previous studies acquired BS2 from the same source (Animal Services Unit of the University of Bristol), the discrepancy in findings may be due to the variability of traits between the generations of inbred strain as suggested by Wong *et al.* [53]. Most of the traits or phenotypes of inbred strains are well conserved throughout the lineage. However, there are a few factors (e.g., genetic, environment) that may influence and alter certain phenotypes from one generation to another [53].

We found that BS2 guinea pigs, like other animal controls, were not protected from developing OA. There was no previous report of when the earliest cartilage damage appeared in this strain. In this study, the fibrillated cartilage was seen in most BS2 guinea pigs as early as 24 weeks (except in 1 animal which showed fibrillation at 10 and 16 weeks). In this study, at 30 weeks of age, the severity of cartilage degradation in the heavier DH strain was significantly higher than the BS2, suggesting that at least at the late stage disease, body weight influences cartilage severity. Similarly, a study on restriction of food intake in DH showed that 65% of food restriction can reduce the severity of AC damage by 40% [54]. Taken together, the above findings suggest that body weight plays an important role in the progression of OA, and that it may partly explain why OA developed earlier and progressed faster in the heavier DH than the BS2 strain. Weiser-Maples guinea pigs used as controls in another study were also significantly lighter than DH but they did not show any fibrillation at least up to 32 weeks of age [35]. In contrast, strain 13 used by Huebner *et al.* [34] as control had only a minimal cartilage degradation and PG loss by 12 months of age. Due to the fact that Strain 13 did not have body weight differences with DH for the first 6 months and developed OA later than BS2 or Weiser-Maples, in future studies Strain 13 would be a better choice of control for DH strain.

This study has several limitations. First, lack of significant differences in chondrocyte apoptosis between strains may be simply due to limited numbers of animals per time point. Secondly, the control strain (BS2) used in the study also developed OA although at slower rate, therefore whether chondrocyte apoptosis is involved in initiation of the disease process in DH guinea pigs could not be determined.

## 4. Materials and Methods

### 4.1. Animals

Twenty four male Dunkin Hartley (DH) guinea pigs were purchased at 9 weeks of age (Harlan Laboratories Ltd., Oxon, UK), while Bristol Strain 2 (BS2) guinea pigs were inbred at the Animal Services Unit of the University of Bristol. The animals were housed individually/paired in 23 × 52 × 72 cm cages, maintained in a 12 h-lighting/22 °C controlled room, and fed with standard guinea pigs diet (Harlan Teklad, Cheshire, UK) and water *ad libitum*. Vitamin C (1000 mg) was also given by adding between half and one tablet per 500 mL of drinking water.

Body weight of each animal was measured prior to euthanasia at 10, 16, 24 and 30 weeks of age. Six animals from each strain were sacrificed at each time points by an intraperitoneal injection of euthatal solution. The right knee joints were then dislocated and the tibias were collected for histological and immunohistochemical analyses. All the above procedures were carried out in accordance with ethical guidelines approved by our institute (Win No. UB/10/024) and the UK Animals (Scientific Procedures) Act 1986.

### 4.2. Histology and Microscopic Scoring

Frontal sections of un-decalcified proximal tibial epiphysis were cut at 7 μm of thickness using a disposable tungsten carbide blade (Jung TC-65, Leica microsystem) according to a modified application of Kawamoto’s film method [55]. The sections were then stained with toluidine blue and scored blind based on Osteoarthritis Research Society International (OARSI) recommended scoring scheme for guinea pigs [56]. This scoring system grades AC based on five main criteria namely AC structure, PG content, cellularity, tidemark intergrity and osteophyte formation. Due to sampling limitations, the scores for osteophyte formation were omitted, thus each individual section would have a maximum total score of 18, instead of 21.

### 4.3. Caspase-3 Staining

Caspase-3 expression by apoptotic chondrocytes was detected by immunostaining technique as previously described with modification [3]. Briefly, the histological sections were fixed in 4% formaldehyde prior to blocking with a goat serum (1:5, Vector). The sections were then incubated with the primary antibody against active caspase-3 (1:200, rabbit polyconal, Minneapolis, MN, USA; R & D systems, Abingdon, UK) and followed by the biotin-labelled secondary antibody (1:200, goat anti-rabbit, Milipore, Massachusetts, MA, USA) for 1 h each. The negative control section was incubated with PBS alone, without the primary antibody. Following this, an extra-avidin alkaline phosphatase conjugate (1:100, Sigma, Dorset, UK) was applied at a dilution of 1:100. After 30 min of incubation, fast red substrates (Sigma, Dorset, UK) was added and left to incubate for 10 min before being counterstained with Carazzi’s haematoxylin (Section Lab, Hiroshima, Japan). The stained sections were examined and the number of caspase-3 positive/negative chondrocytes was counted under a light microscope at ×400 magnification by an observer who was blinded to the age and strain of the animals. The percentage of chondrocytes undergoing apoptosis in the medial and lateral side of each section was calculated separately by dividing the number of positive staining chondrocytes with the total number of chondrocytes in the respective side.

### 4.4. Terminal Deoxyribonucleotidyl Transferase (TdT)-Mediated dUTP Nick-End Labelling (TUNEL) Staining

TUNEL is an established technique for detecting apoptotic chondrocytes by labelling the fragmented DNA with deoxyribonucleotide triphosphate (dNTP). TUNEL was performed on randomly selected sections from each strain (DH: *n* = 4; BS2: *n* = 4) using a TACS^®^ 2 TdT-DAB *in situ* detection kit (R & D systems, Abingdon, UK). For the negative control, the section was incubated with the labelling buffer alone whilst the positive control section (Tissue control slide, R&D system, Abingdon, UK) was treated as described by the manufacturer.

### 4.5. Statistics

PASW Statistics 18 software was used for statistical analysis. The data were tested for normality using Kolmogorov-Smirnov test. Wilcoxon signed-rank test, Mann Whitney U test and Kruskal-Wallis test (followed by Bonferroni post-hoc test) were used to compare the data between sides (medial *versus* lateral condyle), animal strains (DH *versus* BS2), and time points, respectively. Correlation between the percentage of chondrocyte apoptosis and each component of microscopic scores was tested by using Spearman’s correlation test. The data were presented as mean (95% confidence interval (CI)) and a significant difference of *p* ≤ 0.05 and *p* ≤ 0.01 was denoted as * and **, respectively.

## 5. Conclusions

In summary, our data show that progression of chondrocyte apoptosis follows cellularity changes in the medial side of AC, suggesting that cell death is a late event in cartilage changes in OA. Moreover, the data also suggest that body weight is a risk factor for progression of apoptosis and cartilage degradation. In addition, localisation of apoptotic chondrocytes in the DZ of cartilage may indicate its association with subchondral bone remodelling during the development of OA, which is an important area of our future investigation. Since the BS2 strain developed OA earlier than expected, its reliability as a control for the OA prone DH strain is uncertain.
